# A bespoke health risk assessment methodology for the radiation protection of astronauts

**DOI:** 10.1007/s00411-021-00910-0

**Published:** 2021-04-30

**Authors:** Linda Walsh, Luana Hafner, Ulrich Straube, Alexander Ulanowski, Anna Fogtman, Marco Durante, Guillaume Weerts, Uwe Schneider

**Affiliations:** 1 Department of Physics, Science Faculty, University of Zürich, Winterthurerstrasse 190, 8057 Zurich, Switzerland; 2 Department of Physics, ETH Zurich, Otto-Stern-Weg 1, 8092 Zurich, Switzerland; 3 Medical Operations and Space Medicine, HRE-OM, European Space Agency, ESA, European Astronaut Centre, EAC, Cologne, Germany; 4 Present Address: Environment Laboratories, International Atomic Energy Agency, 2444 Seibersdorf, Austria; 5 Institute of Radiation Medicine, Helmholtz Zentrum München- German Research Center for Environmental Health, 85764 Neuherberg, Germany; 6 Biophysics Department, GSI Helmholtzzentrum für Schwerionenforschung, Darmstadt, Germany; 7 Technische Universität Darmstadt, Darmstadt, Germany; 8 Radiotherapy Hirslanden, Witellikerstrasse 40, 8032 Zurich, Switzerland

**Keywords:** Space radiation protection, Space flight, Radiation-related cancer, Radiation attributed decrease of survival, Radiation risk model

## Abstract

An alternative approach that is particularly suitable for the radiation health risk assessment (HRA) of astronauts is presented. The quantity, Radiation Attributed Decrease of Survival (RADS), representing the cumulative decrease in the unknown survival curve at a certain attained age, due to the radiation exposure at an earlier age, forms the basis for this alternative approach. Results are provided for all solid cancer plus leukemia incidence RADS from estimated doses from theoretical radiation exposures accumulated during long-term missions to the Moon or Mars. For example, it is shown that a 1000-day Mars exploration mission with a hypothetical mission effective dose of 1.07 Sv at typical astronaut ages around 40 years old, will result in the probability of surviving free of all types of solid cancer and leukemia until retirement age (65 years) being reduced by 4.2% (95% CI 3.2; 5.3) for males and 5.8% (95% CI 4.8; 7.0) for females. RADS dose–responses are given, for the outcomes for incidence of all solid cancer, leukemia, lung and female breast cancer. Results showing how RADS varies with age at exposure, attained age and other factors are also presented. The advantages of this alternative approach, over currently applied methodologies for the long-term radiation protection of astronauts after mission exposures, are presented with example calculations applicable to European astronaut occupational HRA. Some tentative suggestions for new types of occupational risk limits for space missions are given while acknowledging that the setting of astronaut radiation-related risk limits will ultimately be decided by the Space Agencies. Suggestions are provided for further work which builds on and extends this new HRA approach, e.g., by eventually including non-cancer effects and detailed space dosimetry.

## Introduction

Uncertainties in detrimental health risks from space radiation exposure are main mission-duration limiting factors in the planning of long-term interplanetary missions involving astronauts (Hellweg et al. 2020; Chancellor et al. 2014). Current detrimental health risk assessments for space applications mainly consider how to account for and protect against heavy-ion carcinogenesis (Durante and Cucinotta 2008). Different national space agencies currently adopt non-aligned approaches for limiting the total radiation exposure accumulated during the career of an astronaut and this non-alignment poses problems for planning international exploratory-class missions (McKenna-Lawlor et al. 2014).

A recent position paper, on research plans in Europe for radiation hazard assessments in space, recommended the development of European space radiation risk models to better characterize the radiation risks to astronauts (Walsh et al. 2019a). In line with this recommendation, an alternative approach to the radiation health risk assessment (HRA) of astronauts and the advantages of this approach, over currently applied methodologies for astronaut HRA, are presented here. Application of a cumulative risk measure called Radiation Attributed Decrease of Survival (RADS) and representing the cumulative decrease in the unknown survival curve at a certain attained age, due to the radiation exposure at an earlier age, forms the basis for this alternative approach (Ulanowski et al. 2019, 2020a). This risk assessment approach is somewhat simpler than currently applied radiation lifetime risk measure methodologies, and relies less, than current methodologies, on detrimental health information drawn from the general population, which are not a good proxy for atypically healthy, non-smoking and carefully health monitored astronauts.

In comparison to other lifetime cumulative risk measures (Vaeth and Pierce 1990) such as Life Attributable Risk or Years of Life Lost/Relative Reduction of Lifetime and the Risk of Exposure Induced Death (REID), where this latter quantity is currently applied in the US for astronaut radiation HRA (NASA 2007, 2013), RADS requires fewer assumptions and input parameters, making a comprehensive uncertainty analysis more realistically achievable than in the past. A systematic overview of the conventional and alternative risk metrics is beyond the scope of this study and can be found in Ulanowski et al. (2020a, b). The outcomes for disease incidence groupings rather than disease mortality groupings has been applied in this approach for the many reasons given in the discussion section. However, RADS is a generic term for a quantity which may be applied either to disease mortality outcomes or to disease incidence outcomes. When applied to disease incidence outcomes, RADS represents the radiation attributed decrease in disease-free survival.

This alternative approach is presented here for several sex-specific disease incidence outcome groupings: all solid cancer plus leukemia, all solid cancer, leukemia, lung cancer and female breast cancer. For comparison purposes, some of the results for RADS are compared here directly to results computed for the parallel incidence quantity to REID, i.e., Risk of Exposure Induced Cancer (REIC), using European population data and applying the same framework to both risk quantities. All of the cumulative risk quantities mentioned need to be based on radiation-related disease risk models from existing radiation epidemiology cohorts with long-term follow-up. Currently, risks (e.g., Grant et al. 2017) based on the Life Span Study (LSS) cohort data from survivors of the A-bombs in Japan, exposed to gamma and neutron radiation, are applied for this purpose. Such risk models give the disease risks per unit weighted or equivalent organ dose, i.e., weighted by the A-bomb neutron Relative (to gamma) Biological Effectiveness (RBE), so the choice of this LSS specific neutron RBE directly affects the risk estimates per unit weighted organ dose calculated from the A-bomb epidemiological data. LSS neutron RBEs higher than the value of 10, generally applied, result in smaller LSS based risks per unit weighted or equivalent A-bomb organ dose. For this reason, a clear distinction is required here between the neutron RBE, which is by necessity applied in obtaining the risk models from the A-bomb survivors (LSS neutron RBE), and the neutron RBE (and other RBE types) appropriate for the neutron fluence of galactic cosmic radiation and solar radiation (space RBEs).

Recent indications (Cordova and Cullings 2019, and earlier references cited therein) of higher LSS neutron RBEs than 10, are taken into account in the alternative approach by applying a published empirical LSS neutron RBE model (Hafner et al. 2021). The LSS neutron RBE is not directly applicable to astronaut dosimetric monitoring data as a proxy for neutron space RBE. Ultimately, however, such A-bomb-based risks per unit weighted LSS organ dose should be applied to the unit weighted organ doses calculated from actual astronaut dosimetry monitoring data and appropriate space RBEs for different space radiation types taken from authoritative assessments (e.g., ICRP 2013). It is these weighted or equivalent organ doses from the space radiation that need to be applied in the future, within the current HRA framework, to obtain the actual risks for astronauts. However, the inclusion of actual astronaut dosimetry monitoring data and detailed space dosimetry is beyond the scope of the current paper.

To illustrate the application scope of this alternative HRA methodology, risks are presented at: either a 1 Gy weighted A-bomb organ dose or dose response; or for estimated effective doses for several hypothetical Moon or Mars missions. For this purpose, assumptions are made that different unit organ doses in Gy (i.e., for colon and bone marrow) will be equal and also equal to the effective dose in Sv, for very high energy space radiation and for a given fluence, as first-order approximations.

Further work, described in the discussion section, will be done in the future to include actual astronaut dosimetry monitoring data and to assess the feasibility of including other important detrimental health outcomes such as cardiovascular and central nervous system diseases into this RADS-based HRA methodology.

## Materials and methods

### General description of the methods

Most currently applied cumulative lifetime measures for assessing the risk of radiation-related detrimental health outcomes (e.g., incidence or mortality in various cancer groupings), are based on the radiation risks from the Life Span Study (LSS) of Hiroshima and Nagasaki A-bomb survivors (e.g., Preston et al. 2007; Grant et al. 2017). The LSS provides data for fitting mathematical models for the proportional (excess relative risk, ERR) and additive (excess absolute risk, EAR) increases of the radiation-related rates of the outcome of interest (cancer or other disease) with respect to the LSS baseline rates. These risks are usually expressed as a function of weighted unit organ dose from the A-bombs (LSS gamma organ dose + 10 × LSS neutron organ dose) appropriate for the outcome considered, assuming an LSS neutron RBE of 10. Also, a risk centering at an age at exposure (*e*) of 30 years and attained age (*a*) of 70 years (i.e., age-centred sex-averaged risks) years is generally used for various models for different cancer groupings (e.g., all solid cancer). The LSS risk models are completed with risk effect modifiers that can be applied to re-calculate these central risks to provide risks at different ages at exposure, attained ages and for males and females.

In radiation health risk assessment, it is often required, not only to know the risks at certain ages and not only with respect to the LSS baseline rates in various cancer groupings but to have a measure of the lifetime (or segment of lifetime) risks after radiation exposure in different populations. To calculate such cumulative risks, the ERR and EAR from the LSS (or other radiation epidemiology studies) are usually combined with the baseline rates in the population of interest, and the general survival characteristics in the population of interest and integrated over age, from age at exposure (accounting for outcome latency time) up to the age at which the cumulative risk is required. Vaeth and Pierce (1990), introduced several cumulative risk measures generally applied in radiation health risk assessments, including the Risk of Exposure Induced Death (REID), as currently applied by NASA. REID is based on the survival function, i.e., the dose-dependent conditional survival probabilities of persons alive at age at exposure, *e*, to reach at least age *a*. This survival function, of the exposed population, includes acute mortality after exposures up to several Gy and late radiation-induced non-cancer mortality (Kellerer et al. 2001), where these latter two quantities have very large uncertainties.

In general, uncertainties are high when lifetime cumulative risks are based on survival functions that need to be projected far into the future, because (a) the current population disease and death rates must be assumed to remain constant far into the future (Walsh et al. 2014) and (b) the survival characteristics of the at-risk exposed persons (i.e., astronauts here) must be assumed to be well represented by the general population. A radiation risk assessment method, that inherently substantially reduces the dependence on population statistics and completely removes the requirement of survival curve input, has recently been described (Ulanowski et al. 2019). This method, called radiation attributed decrease of survival (RADS) is particularly useful for risk assessments in highly atypical exposed groups such as astronauts (i.e., atypically healthy, non-smoking and carefully health monitored) and forms the basis of the work presented here for a bespoke approach to HRA for the radiation protection of astronauts (see also Walsh et al. 2019b). The first stage in this approach will be based on outcome incidence rather than mortality and a consideration of the LSS neutron RBE, inherent in the LSS A-bomb radiation risk models, will also be included. RADS represents a cumulative radiation risk which is only based on the radiation-attributed hazard, *H*_c_, which is just the total integrated excess incidence risks for the outcome of interest, *c*, and is conditional on survival until a certain age $a$:

1 $$\mathrm{RADS}\left(a|e,D,\mathrm{RBE}\right)=1-\mathrm{exp}\left(-{H}_{\mathrm{c}}\left(a|e,D,\mathrm{RBE}\right)\right),$$

where *D* is the organ weighted dose and RBE is the LSS neutron relative biological effectiveness. For example, for the outcome of all solid cancer, *s*, and colon dose, *D*_c_, the hazard is:

2 $${H}_{\mathrm{s}}\left(a|e,D,\mathrm{RBE}\right)= {\int }_{e+l}^{a}{h}_{\mathrm{s}}\left(u,e,{D}_{\mathrm{c}},\mathrm{RBE}\right)\mathrm{d}u.$$

i.e., an integral from age at exposure plus some latency period, l, up to any required attained age, where

3 $${h}_{\mathrm{S}}\left(a,e,{D}_{\mathrm{c}},\mathrm{RBE}\right)=\frac{w{\mathrm{ERR}}_{\mathrm{S}}\left({D}_{\mathrm{c}},a,e,\mathrm{RBE}\right){m}_{S}\left(a\right)+(1-w){\mathrm{EAR}}_{S}({D}_{\mathrm{c}},a,e,\mathrm{RBE})/\mathrm{10,000}}{\mathrm{DDREF}}$$

which accounts for a dose and dose rate effectiveness factor (DDREF) and population age and sex-specific cancer incidence rates *m*_s_(*a*). The factor *w* is applied to weight the relative contributions by the ERR and EAR models. ICRP (2007) stated that weights of 0.5 were applied in detriment calculations, except for the outcomes breast cancer and leukemia, where only an EAR model was applied and except for lung cancer where a *w* = 0.3 was applied—these are the weights adopted in the results section except for leukaemia where a weight of 0.5 was applied also for the outcome all solid cancer plus leukemia. Although, to reflect the full range in the uncertainty connected with the choice of numerical values for w, the results for incidence risks in various cancer groupings are presented here also with the extreme values of *w* = 0 and *w* = 1. For the outcome leukemia, the application of a DDREF is not recommended (ICRP 2007) and, therefore, not applied here.

### Input requirements for calculating RADS

1. Outcome types initially considered. The following malignant diseases or groups of malignant diseases were initially considered (ICD-10 classification codes are shown in parentheses) because they are known to be important sites for adult HRA (ICRP 2007):
2. All solid cancers (C00–C80);
3. Leukemia, defined here as all leukemia (i.e., most of the ICD10: C91–C95 subclasses, excluding CLL, C91.1 and C91.4, and excluding ATL, C91.5);
4. Female breast cancer (C50);
5. Lung cancer (C34).
6. Population age-specific cancer incidence rates, given by sex, cancer site and 5-year age group, are available from the Cancer Incidence in Five Continents Volume XI (CI5 XI) (Bray et al. 2017). The rates for the regions in the following eight countries were combined: Belgium, Denmark, France, Germany, Italy, The Netherlands, Spain, United Kingdom, because this was assumed to be a good representation of European rates for European Space Agency (ESA) astronauts. Originally, Sweden was in the choice of countries to combine but the Swedish rates were not included in CI5 XI. Combined rates for these 8 countries were obtained by first sorting by Continent, to exclude colonies outside Europe, and calculated by taking the mean of incidence rates from the years 2008–2012 for the following cancers: (ICD-10 code) breast (C50), lung (incl. trachea and bronchus) (C33–34), lymphoid leukemia (C91), myeloid leukemia (C92–94), leukemia unspecified (C95). Results are also presented for all solid cancer plus leukemia with the combined ICD-10 code as already given for all solid cancer and leukemia separately. These ICD groups match the ICD 10 codes pertaining to the LSS cancer risk models, as closely as possible.
7. The LSS ERR model for all solid cancer based on colon dose from Grant et al. (2017) and the EAR model for all solid cancer from Walsh et al. (2019b) were applied. LSS ERR and EAR models for leukemia based on red bone marrow dose from Hsu et al. (2013) were applied. For female breast cancer models based on breast dose from Preston et al. (2007) were applied. For lung cancer, the radiation only ERR model from Table 3 of Cahoon et al. (2017) and an EAR model fitted, by the current authors, by changing this ERR into an additive model with otherwise the same parameterisation, based on lung weighted organ dose were applied. These model choices were based on models fitted to the most recently available LSS incidence data for which the data and EPICURE scripts were publicly available because we needed to re-fit the same models, with the extra information on the (unavailable) parameter covariance matrices that we needed to apply in the uncertainty analysis. All implemented ERR and EAR model parameters are given in Table 4 of Appendix A.
8. The dose and dose rate effectiveness factor (DDREF). The current methodology allows a user-based choice in the software developed for this HRA (see below) of either an ICRP (2007) recommended value of a DDREF = 2, or any other value. The impact of uncertainties in the DDREF can be assessed by running the prototype risk calculation software with different values. This choice can eventually be made, and uncertainties included, to reflect the future recommendations for DDREF from ICRP Task Group (TG) 91 on “Radiation Risk Inference at Low-dose and Low-dose Rate Exposure for Radiological Protection Purposes”. This TG is currently evaluating the scientific evidence of low-dose and low-dose-rate effects (Rühm et al. 2015, 2016, 2018; Shore et al. 2017). However, all results presented here are for a DDREF of 1, for the purposes of illustrating this HRA methodology. For the outcome leukemia, a DDREF is not applied here in line with ICRP recommendations (ICRP 2007). For missions with organ doses above the usual levels considered in ground-based radiation protection of up to a few hundred mGy, it may be more appropriate to call this factor a dose rate effectiveness factor (DREF) (see “Discussion” section).
9. The LSS neutron RBE. The impact, of different A-bomb neutron RBEs assumed in the LSS risk models, on radiation risk models applied in space HRA has already been considered (Schneider and Walsh 2009). The current methodology allows a user-based choice because this previous work has been extended and an LSS neutron RBE model has been recently developed (Hafner et al. 2021) and applied here. An LSS neutron RBE value of 80 is applied here for the purposes of illustrating this HRA methodology, since Cordova and Cullings (2019) and other comprehensive studies cited therein, found indications that the LSS neutron RBE for colon dose is 80 (95% CI 20; 190), i.e., much higher than the value of 10 usually applied in most official analyses of the LSS data. The impact of uncertainties in the LSS neutron RBE can be assessed by running the prototype risk calculation software with different values.
10. Mission-based data on the astronauts at risk. The current software implementing this HRA methodology allows a user-based choice of the number of missions (1 up to a current maximum of 4) and for each mission, the astronauts age in years and organ dose for the chosen outcome of interest i.e., colon dose, red bone marrow (RBM) dose, breast dose and lung dose for the outcomes all solid cancer, leukemia, female breast cancer and lung cancer, respectively. The results for all solid cancer plus leukemia risk are calculated by either: adjusting the RADS outcome to “all solid cancer and leukemia” before integration, Eq. (2); or adding the REIC risks per unit organ dose for both outcomes—both procedures are based on the assumption that colon and the RBM organ doses in Gy will be equal and equal to the effective dose in Sv, for very high energy space radiation for a given fluence, as a first-order approximation. For the purposes of illustrating the HRA methodology, results are presented for either a 1 Sv dose or a dose response or for approximate characteristic doses for several hypothetical Moon or Mars missions resulting in doses of 0.17, 1.03 or 1.07 Sv, estimated at solar minimum and with a 5 g cm^−2^ aluminium shielding of the space craft (NASA 2005; Cucinotta 2007).
11. An accounting for the non-smoking status of astronauts is required because CI5 XI population age-specific lung cancer incidence rates *m*_s_(*a*), applied in Eq. (3), are generally only available in Europe for the general population [made up from all types of smoking behaviour: current smokers (CS); never smokers (NS); and former smokers (FS)]. The NASA methodology (Chappell 2014) has been adopted, adapted and applied here to European CI5 XI data to estimate NS cancer incidence rates from the following: relative risk for lung cancer mortality associated with smoking exposure in CS and FS relative to never smokers (Thun et al. 2013); A lethality factor for lung cancer of 0.89 for males and females (ICRP 2007); and the prevalence of NS, FS and CS in the German population for 2012 (from Figs. 2 and 3 of Zeiher et al. 2018). This latter source of prevalence information was applied under the assumption that prevalence percentages in the German population are a good approximation to prevalence in the 8 European countries combined CI5 XI data. Appendix B gives further details of this methodology.
12. Choices for latency periods, *l*, to apply in Eq. (2) above, for each outcome are required. Here the latency periods applied in the WHO Fukushima HRA of 2 years for leukemia and 5 years for the other outcomes considered here, were adopted (WHO 2013; Walsh et al. 2014).

### Input requirements for calculating REIC

The full methodology and equations for calculating REIC have already been given and results for all solid cancer plus leukemia have already been provided for astronaut HRA (Hafner et al. 2021). The only differences between the calculations provided for REIC in Hafner et al. (2021) and here are that in Hafner et al. (2021), the German cancer incidence rates were applied instead of the CI5 XI—eight country rates used here. The other input requirements 1–8 given above, are actually the same for REIC and for RADS, but with the additional requirement for REIC of a dose-dependent survival curve that accounts for late radiation-induced non-cancer mortality, such as cardiac mortality and acute radiation-induced all-cause mortality with the model forms already given in detail by Hafner et al. (2021). The large uncertainties in the survival curve coming from, among other sources, the uncertainties in the models accounting for late and acute mortality, were not accounted for here because these models are only published with parameter central estimates.

### The software applied and the accounting for uncertainties

Published ERR and EAR models were refitted by the current authors to obtain the model fit-parameters (Table 4 of Appendix A) and the parameter covariance matrices (Tables 5, 6, 7, 8, 9, 10, 11 and 12 of Appendix A) using the EPICURE software with the AMFIT module (Preston et al. 1993). The RADS methodology included a Monte-Carlo uncertainty analysis and was programmed in the open-source R-statistical programming language, making use of the open-source R-function called mvrnorm for Monte-Carlo simulations accounting for correlations. A user-friendly graphical interface (programmed in SHINY R graphical interface, https://shiny.rstudio.com) was created allowing for different choices of input parameters (e.g., DDREF, LSS neutron RBE, number of realisations, etc.) and either graphical or tabular output of the HRA results. The Monte-Carlo simulations were done to account for the uncertainties of the ERR and EAR fit parameters in the calculation of RADS, by computing new sets of realisation of these parameters by sampling according to the parameter covariance matrices. Uncertainties in the European 8-country CI5 XI baseline incidence rates were considered by a Poisson sampling of the rates. The number of realisations used was either 1000 for each point in the graphical results or 10,000 for the tabular results. The Monte Carlo simulations were used to provide 95% confidence intervals (for modelling uncertainty) on RADS and REIC applied here as the applicable uncertainty range for all the results. The simple Newton–Cotes rectangle rule method was applied for the numerical integrations over age, in one-year intervals of age. On repeated calculations with 10,000 realisations, the 95% CI values on RADS were stable to within ± 0.02%.

### Application of risk limits

Since RADS is free from assumptions on future survival, it is not limited in time and, following the underlying model’s prediction, has a strong dependence on the attained age. No official RADS limit has been published so far, so different risk limit settings are tentatively suggested here. In general, risk limits are related to limits on numbers of fatality cases, within a pre-defined time frame. However, in this application, risks for various cancer groupings based on incidence rates and incidence models are applied, so a transfer of mortality risk limits to incidence may be required for radiation protection discussions and decisions related to the feasibilities of potential space missions.

There are several ways to approach the setting of limits and two suggestions are given here. NASA applies a limit of 3% at the 95% upper confidence level of the risk of exposure-induced death (REID) (NASA 2013). A general lethality factor of 0.49 results for all cancers using different cancer lethality factors (taken from Table A.4.5 of ICRP 2007) when weighted with the cancer case counts of the A-bomb survivor data. Taking this particular general lethality factor into account, the incidence limit for REIC and RADS corresponding to the NASA mortality limit could be set to be 6.1% at the upper 95% CI.

The ICRP (1991) report states that one case of death per year per 1000 persons is acceptable as a measure for occupational risk. This corresponds to a fatality risk of 0.1% per year and a total occupational fatality risk of 4%, assuming a work life of 40 years and a retirement age of 65 years. Consequently, another possibility could be to set the 95% limit for RADS to 4% lethality factor. This will lead to a limit of 8.2% which, for a working life can be assumed to remain constant at this level for ages above 65 years for all solid cancer plus leukemia incidence. Using the fatality risk of 0.1% mentioned above, a factor of 0.2% per year could then be subtracted from the 8.2% for each age below 65 years. Risk limits could be chosen to pertain to any period post-exposure, i.e., the rest of lifetime (as with current NASA limits) or a total occupational risk limit up to retirement age of e.g., 65 years of age or a composite limit e.g., RADS at age 65 years (for retirement planning) considered in tandem with RADS at age 80 years (for lifestyles planning at advanced ages).

## Results

All risk results presented in this section are for various cancer incidence groupings and given with 95% confidence intervals calculated with Monte-Carlo simulations. A DDREF of 1 was applied consistently in all results. The LSS ERR and EAR models, that go into the calculation of RADS, were consistently adjusted to account for an A-bomb fission neutron RBE relative to gammas, in the LSS data of 80. This choice of 80, can be adjusted in the software to other values, is specific to the A-bomb risk models and should not be interpreted as a suggestion to apply a neutron RBE = 80 for neutron components of space radiation. No baseline scaling between LSS baselines and CI5, 8-country baseline rates was applied for reasons already given (Hafner et al. 2021, which also contains results both with and without this type of scaling).

### Comparisons of REIC and RADS for all solid cancer plus leukemia incidence

**Fig. 1:**
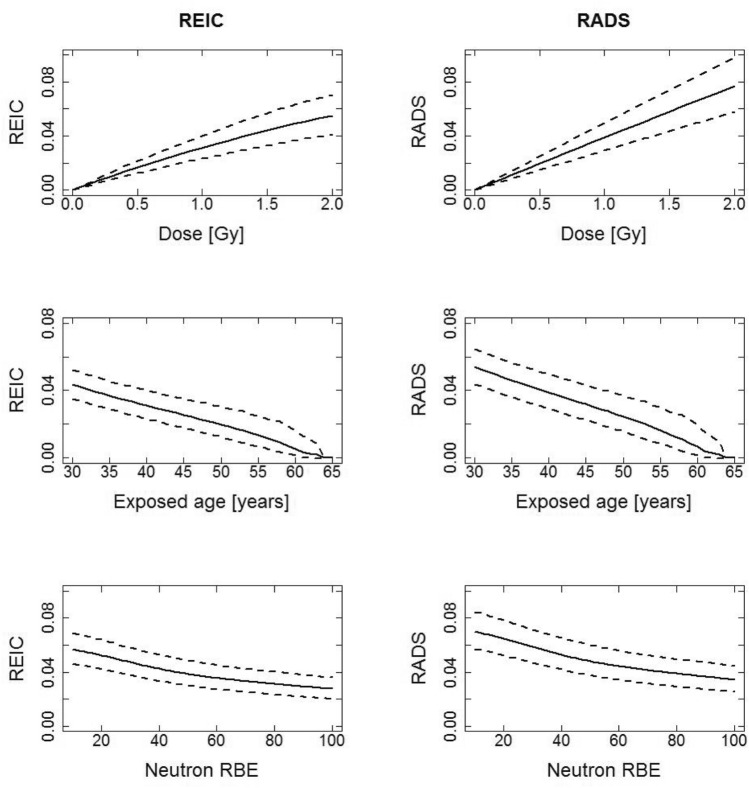
Comparison of REIC and RADS, in decimals, for males for all solid cancer plus leukemia using age at exposure of 40, an attained age of 65, an organ dose of 1 Gy and equal weighting for the ERR and EAR in all solid cancer model and in the leukemia model. It is assumed here that the effective dose, *E* in Sv is equal to the organ dose in Gy. Note: the very large uncertainties in the survival curve from e.g., the radiation dependant acute mortality in the survival curves above 2 Gy, are not included in the results for REIC

**Fig. 2:**
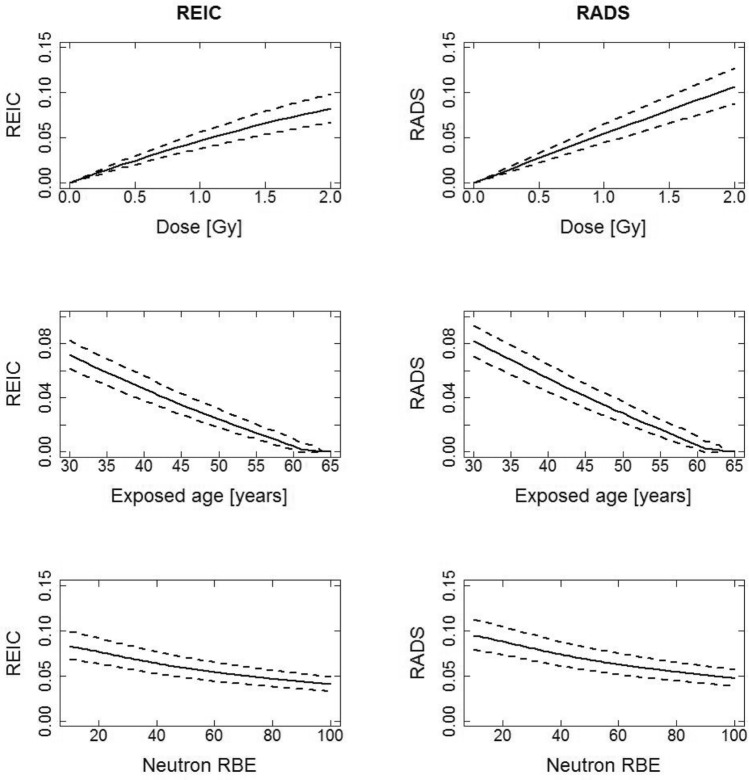
Comparison of REIC and RADS, in decimals, for females for all solid cancer plus leukemia using age at exposure of 40, an attained age of 65, an organ dose of 1 Gy and equal weighting for the ERR and EAR all solid cancer model and leukemia model. It is assumed here that the effective dose, *E* in Sv is equal to the organ dose in Gy. Note: the very large uncertainties in the survival curve from e.g., the radiation dependant acute mortality in the survival curves above 2 Gy, are not included in the results for REIC

Figures 1 and 2 for males and females respectively, show REIC and RADS for all solid cancer plus leukemia incidence, calculated with a DDREF of 1 for the all solid cancer contribution and an LSS neutron RBE of 80 applied to the LSS organ doses unless specified in the x-axis. Figures 1 and 2 show 3 types of plots for each risk type (REIC and RADS): plot type 1—the functional behaviours of risk at attained age 65 year after exposure at age 40 years to different organ doses up to 2 Gy (the risk to dose response); plot type 2—the risk at attained age 65 as a function of age at exposure to 1 Gy organ dose; plot type 3—the risk at attained age 65 years, as a function of LSS neutron RBE after exposure at age 40 years to 1 Gy organ dose. These figures are given with 95% confidence intervals as uncertainty ranges (dashed lines) and for an equally weighted (*w* = 0.5) contribution of ERR and EAR in the all solid cancer model and in the leukemia model. The first row of plots (type 1) in Figs. 1 and 2 shows that the risks for females are higher than the risks for males. At the doses under about the 2 Gy organ dose limit shown, the REIC and RADS risks are very similar. The second row of plots (type 2) in Figs. 1 and 2 shows how the risks decrease with age at exposure, again the REIC and RADS risks show very similar trends. The third row of plots (type 3) in Figs. 1 and 2 shows how the risks decrease with increasing assumed LSS neutron RBE, again the REIC and RADS risks show very similar trends. As previously noted by Hafner et al. (2021) for REIC and by Schneider and Walsh (2009) for REID, a higher neutron RBE applied to the LSS organ doses than the value of 10, traditionally used in the LSS risk models, can reduce the cumulative risks up to almost 50%, and this trend is also seen for RADS here. To help a reader who may be more familiar in interpreting cumulative risk estimates obtained using LSS risks per unit LSS doses calculated with the LSS neutron RBE of 10, conversion factors, *f*, where RADS (LSS neutron RBE = 10) = *f* × RADS(LSS neutron RBE = 80), for the conditions in Figs. 1 and 2 (plot type 3), are *f* = 1.79 for males and *f* = 1.74 for females. The practical result of this behaviour is that longer space missions may be justifiable within given risk limits of radiological protection.

### Comparisons of REIC and RADS for the outcome all solid cancer plus leukemia incidence for different space missions

In Table 1 REIC and RADS central estimates and 95% CIs are given for different hypothetical space missions. The missions considered are a Lunar mission lasting 180 days with an astronaut exposure to 0.17 Sv effective dose, a 600-day duration Mars swing-by mission for an exposure of 1.03 Sv and a Mars exploration mission lasting 1000 days and resulting in an exposure of 1.07 Sv. The exposures are assumed to occur at an average age at exposure of 40 years and the risks pertain to an attained age of 65 years. The calculations are based on the assumption that the effective dose in Sv is equal to the colon and RBM organ doses in Gy. The RADS central estimate for females is systematically 1.4 times higher than for males. The RADS central estimate lies under the tentatively suggested 6.1 and 8.2% upper 95% CI limits for every scenario, except for the two Mars missions for women at the upper 95% CI level, which at 6.75 and 7.00% for the swing-by and exploration mission respectively, are over the 6.1% limit option but under the 8.2% limit option both tentatively suggested above. However, when the non-cancer risks are included, in future work, both risk limits could be exceeded.

**Table 1:** All solid cancer plus leukemia incidence risks for different space missions calculated for an average age at exposure of 40 years, an attained age of 65 years and equal weighting for the ERR and EAR all solid cancer model and leukemia models

Mission type, duration (days)	*E*/Sv	REIC [%]		RADS [%]	
		Males	Females	Males	Females
Lunar, 180	0.17	0.59 (0.44; 0.75)	0.88 (0.72; 1.07)	0.67 (0.51; 0.85)	0.94 (0.77; 1.14)
Mars swing-by, 600	1.03	3.20 (2.41; 4.10)	4.79 (3.89; 5.82)	4.00 (3.05; 5.11)	5.59 (4.57; 6.75)
Mars exploration, 1000	1.07	3.31 (2.49; 4.24)	4.95 (4.03; 6.02)	4.16 (3.16; 5.30)	5.80 (4.75; 7.00)

It is assumed here that the effective dose, *E* in Sv is equal to the colon and RBM organ dose in Gy, and that the colon organ dose is equal to the RBM dose in Gy. Note: the very large uncertainties in the survival curve from e.g., the radiation dependant acute mortality in the survival curves above 2 Gy, are not included in the results for REIC

### General comparisons of RADS for the four cancer incidence groupings considered

Table 2 gives results for RADS in %, all for an exposure to 1 Gy organ dose at age 40 years, for the four cancer incidence groupings considered and calculated by integrating the Hazard equation, Eq. (3) in the RADS calculation up to different upper limits of attained ages, i.e., 65, 70, 75 and 80 years of age.

**Table 2:** RADS incidence risk for various groupings of cancer incidence risks, in percent, calculated for different attained ages for an exposure to 1 Gy organ dose at age 40 years

Attained age (years)	65		70		75		80	
	Males	Females	Males	Females	Males	Females	Males	Females
Mixed ERR and EAR								
All solid cancer	3.1 (2.5; 3.9)	4.9 (4.1; 5.9)	4.5 (3.6; 5.6)	6.5 (5.4; 7.8)	6.1 (4.9; 7.6)	8.2 (6.9; 9.8)	7.8 (6.3; 9.7)	10.1 (8.5; 12.0)
Leukemia	0.8 (0.1; 1.5)	0.5 (0.1; 1.0)	0.9 (0.2; 1.8)	0.6 (0.1; 1.2)	1.1 (0.2; 2.1)	0.7 (0.1; 1.4)	1.3 (0.2; 2.4)	0.8 (0.1; 1.5)
Lung	0.5 (0.2; 0.7)	0.8 (0.5; 1.3)	0.7 (0.4; 1.1)	1.2 (0.8; 1.7)	1.0 (0.5; 1.5)	1.5 (1.1; 2.2)	1.3 (0.7; 2.0)	2.0 (1.4; 2.7)
Lung adjusted (NS)	0.3 (0.1; 0.5)	0.4 (0.2; 0.5)	0.4 (0.2; 0.7)	0.5 (0.4; 0.8)	0.6 (0.3; 1.0)	0.8 (0.5; 1.1)	0.8 (0.4; 1.4)	1.1 (0.8; 1.5)
Breast	–	0.5 (0.3; 0.8)	–	0.6 (0.4; 1.0)	–	0.8 (0.5; 1.3)	–	1.0 (0.6; 1.6)
ERR (*w* = 1)								
All solid cancer	3.9 (2.8; 5.2)	6.7 (5.2; 8.6)	5.8 (4.2; 7.7)	8.8 (6.8; 11.1)	7.9 (5.8; 10.5)	10.8 (8.4; 13.6)	10.2 (7.5; 13.3)	12.9 (10.1; 16.1)
Leukemia	1.0 (− 0.2; 2.4)	0.7 (− 0.1; 1.7)	1.2 (− 0.2; 2.8)	0.8 (− 0.2; 1.9)	1.5 (− 0.3; 3.3)	0.9 (− 0.2; 2.2)	1.8 (− 0.3; 3.8)	1.1 (− 0.2; 2.5)
Lung	0.8 (0.3; 1.5)	1.8 (1.0; 3.2)	1.1 (0.4; 2.1)	2.4 (1.4; 4.1)	1.5 (0.6; 2.7)	3.1 (1.8; 4.9)	1.9 (0.8; 3.4)	3.6 (2.3; 5.7)
Lung adjusted (NS)	0.1 (0.03; 0.2)	0.2 (0.1; 0.4)	0.1 (0.05; 0.3)	0.4 (0.2; 0.6)	0.2 (0.1; 0.4)	0.6 (0.3; 0.9)	0.3 (0.1; 0.6)	0.8 (0.5; 1.2)
Breast	–	5.0 (2.6; 8.6)	–	6.0 (3.2; 10.1)	–	6.7 (3.6; 11.1)	–	7.4 (3.9; 12.0)
EAR (*w* = 0)								
All solid cancer	2.3 (1.7; 3.1)	3.1 (2.4; 3.9)	3.2 (2.3; 4.3)	4.2 (3.3; 5.3)	4.2 (3.0; 5.6)	5.6 (4.4; 6.9)	5.4 (3.9; 7.2)	7.1 (5.6; 8.8)
Leukemia	0.6 (− 0.01; 1.1)	0.4 (0; 0.8)	0.7 (− 0.01; 1.3)	0.4 (0; 0.9)	0.7 (− 0.01; 1.4)	0.5 (0.0; 1.0)	0.8 (− 0.01; 1.6)	0.5 (0.0; 1.1)
Lung	0.3 (0.1; 0.6)	0.4 (0.2; 0.7)	0.5 (0.2; 0.9)	0.6 (0.4; 0.9)	0.7 (0.3; 1.3)	0.9 (0.6; 1.3)	1.0 (0.4; 1.8)	1.3 (0.8; 1.8)
Lung adjusted (NS)	0.3 (0.1; 0.6)	0.4 (0.2; 0.7)	0.5 (0.2; 0.9)	0.6 (0.4; 0.9)	0.7 (0.3; 1.3)	0.9 (0.6; 1.3)	1.0 (0.4; 1.8)	1.3 (0.8; 1.8)
Breast	–	0.5 (0.3; 0.8)	–	0.6 (0.4; 1.0)	–	0.8 (0.5; 1.3)	–	1.0 (0.6; 1.6)

The weights, *w*, for the mixed ERR and EAR (ERR: EAR) are: all solid cancer (0.5: 0.5), leukemia (0.5: 0.5), lung and lung adjusted for non-smoking (NS) (0.3: 0.7), breast (0: 1)

The top section of this table gives the results calculated with *w* values, used to weight the relative contributions to the hazard, Eq. (3), of the ERR, of 0.5 for all solid cancer and leukemia; 0.3 for lung; and 0 for breast. The middle section of Table 2 shows the effect of using just an ERR weighting (*w* = 1) and the bottom section of this table shows the effect of using just an EAR weighting (*w* = 0). From the top panel of Table 2 it can be seen that the different upper limits of attained ages have a large effect on the RADS central estimates, so that for all solid cancers, the RADS for females at attained age 80 years is 10.1% (95% CI 8.5; 12.0) compared to 4.9% (4.1; 5.9) at attained age 65 years. For males the same type of comparison is 7.8% (6.3; 9.7) at attained age 80 years but 3.1% (2.5; 3.9) at attained age 65 years. So, although the RADS for all solid cancer are generally lower for males than for females (reflecting the trends in the models from the LSS (Grant et al. 2017) used to calculate RADS), the ratio of RADS at attained age 80 to RADS at attained age 65 is larger for males than for females.

The results in Table 2 for leukemia show that the male RADS for all attained ages are higher than for females, reflecting the trends in the models from the LSS (Hsu et al. 2013) forming the basis of the RADS calculations. The opposite trend can be seen for lung cancer where the female risks are between about 1.5–1.7 times higher than for males when the CI5 population lung cancer incidence rates are not adjusted to rates for non-smokers. Once the CI5 lung cancer incidence rates are adjusted to approximate rates for non-smokers (Appendix B), this reduces to about 1.3–1.4, so the female to male ratio of lung cancer RADS is lower in non-smokers than for the general 8-country population (with all types of smoking exposures). If a purely ERR transfer is made (middle section of Table 2) the lung cancer RADS for females is higher than for males by a factor of between 1.9 and 2.6 with mixed smoking exposure CI5 rates or a factor between 1.3 and 1.4 for CI5 rates adjusted to non-smoking.

The RADS for female breast cancer in Table 2 show that the central estimates based on the ICRP (2007) recommended 100% EAR transfer (*w* = 0) are a factor of 10 smaller than with 100% ERR risk transfer at attained age 65 (*w* = 1), with this factor reducing to about 7 at attained age 80 years. The RADS at attained age 80 years is either a factor of 2 or 1.5 times the RADS at attained age 65 years for purely EAR or ERR transfer respectively.

**Fig. 3:**
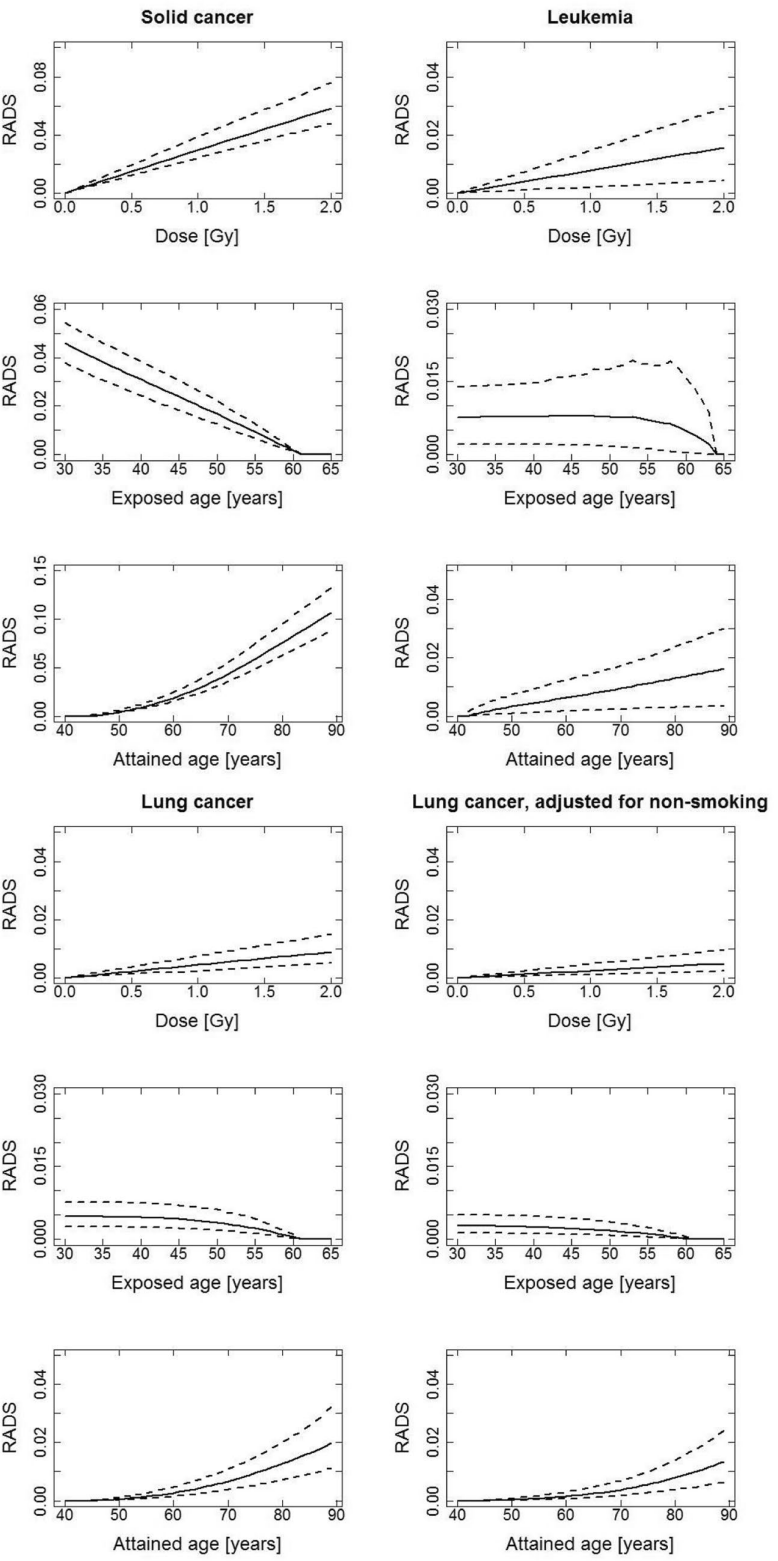
RADS incidence risks for men, in decimals, for different cancer groupings, calculated for an age at exposure of 40 years, an attained age of 65 years and a dose of 1 Gy using a mixed ERR and EAR model. The weights, *w*, for the mixed ERR and EAR (ERR: EAR) are: all solid cancer, abbreviated to solid cancer in the graphics (0.5: 0.5), leukemia (0.5: 0.5), lung and lung adjusted for non-smoking (0.3: 0.7)

**Fig. 4:**
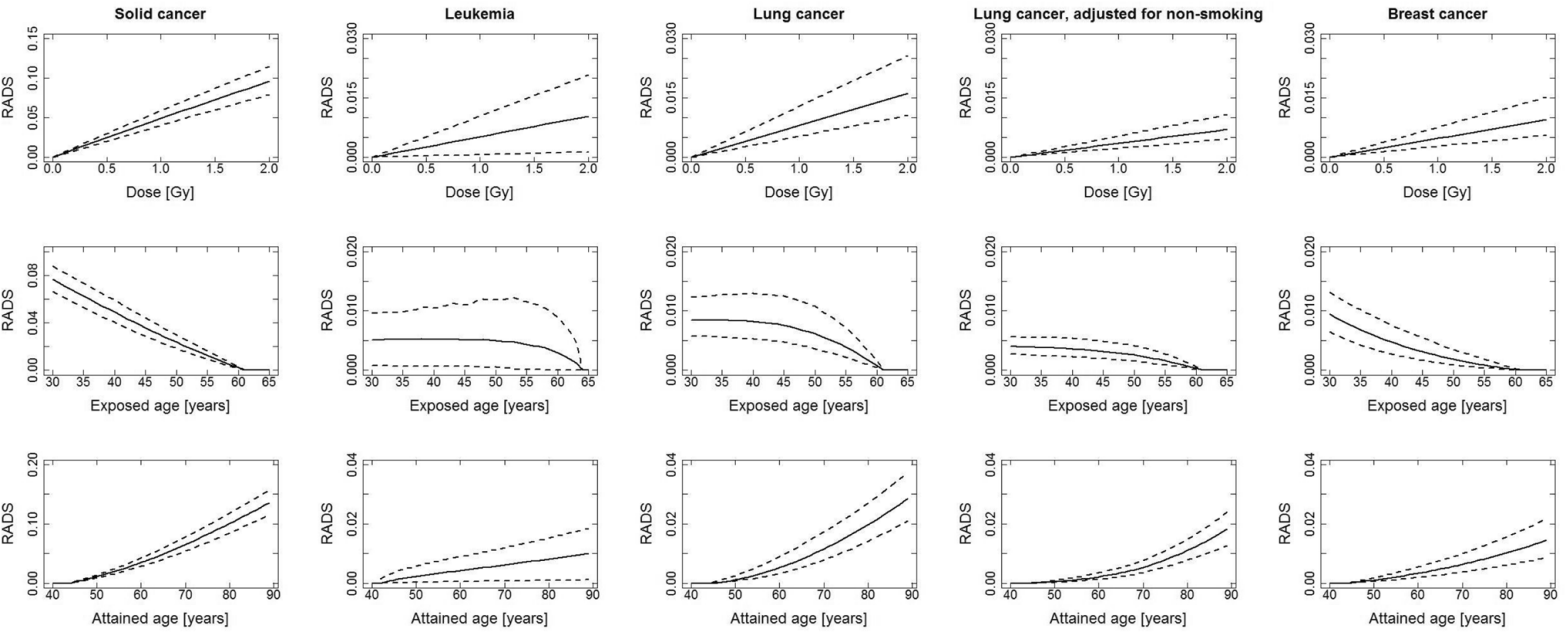
RADS incidence risks for women, in decimals, for different cancer groupings, calculated for an age at exposure of 40 years, an attained age of 65 years and a dose of 1 Gy using a mixed ERR and EAR model. The weights, w, for the mixed ERR and EAR are: (ERR: EAR) all solid cancer, abbreviated to solid cancer in the graphics (0.5: 0.5), leukemia (0.5: 0.5), lung and lung adjusted for non-smoking (0.3: 0.7), breast (0: 1)

Figures 3 and 4, for men and women, respectively, show 3 types of plots for each of the 4 cancer groupings: plot type 1—the functional behaviours of RADS at attained age 65 year after exposure at age 40 years to different organ doses up to 2 Gy (the RADS dose response); plot type 2—RADS at attained age 65 as a function of age at exposure to 1 Gy organ dose; plot type 3—RADS as a function of attained age after exposure at age 40 years to 1 Gy organ dose. Figures 3 and 4 show that the shape of the RADS organ dose response under 2 Gy is linear for all 4 cancer groupings. The dependence of RADS on age at exposure is strongly decreasing for all solid cancers and for breast cancer while remaining quite flat for leukemia and lung cancer. The type 3 plots, for RADS variation with attained age, just reflect the types of results already presented as central estimates in Table 2 and above.

### Comparisons of RADS for different space missions considering the four cancer incidence groupings

Table 3 provides a further breakdown, into cancer groupings, of the RADS for males and females already presented in Table 1. Although RADS for different outcomes is not additive post calculation (see “Discussion” section), it is instructive to be able to see the magnitudes of the relative contributions of the 4 cancer groupings to the RADS for all solid cancer plus leukemia, and the effects on the RADS for lung cancer of applying the CI5 baseline rate adjustment to non-smoking rates. From Table 3 it is clear that leukemia is the second largest contributor to all solid cancer plus leukemia RADS for males and that lung cancer RADS is always a smaller contribution than leukemia for males. For females, leukemia is the second largest contributor to all solid cancer plus leukemia RADS but this contribution is only slightly larger than the contribution from breast cancer. For males and females, the RADS for lung cancer based on the CI5 rates adjusted to rates for non-smokers is numerically smaller than the RADS for the other outcomes.

**Table 3:** RADS incidence risks, in percent, for different cancer sites for different missions

Males	Lunar mission 0.17 Sv	Mars swing-by 1.03 Sv	Mars exploration 1.07 Sv
All solid cancer	0.53 (0.42; 0.67)	3.20 (2.53; 3.99)	3.32 (2.62; 4.14)
Leukemia	0.13 (0.02; 0.26)	0.81 (0.13; 1.56)	0.84 (0.14; 1.62)
All solid cancer plus leukemia	0.67 (0.51; 0.85)	4.00 (3.05; 5.11)	4.16 (3.16; 5.30)
Lung	0.08 (0.04; 0.13)	0.47 (0.25; 0.77)	0.48 (0.26; 0.79)
Lung adjusted (NS)	0.04 (0.02; 0.08)	0.26 (0.12; 0.49)	0.27 (0.12; 0.51)

Females	Lunar mission 0.17 Sv	Mars swing-by 1.03 Sv	Mars exploration 1.07 Sv
All solid cancer	0.85 (0.70; 1.03)	5.06 (4.18; 6.07)	5.25 (4.34; 6.30)
Leukemia	0.09 (0.01; 0.18)	0.54 (0.08; 1.08)	0.56 (0.08; 1.12)
All solid cancer plus leukemia	0.94 (0.77; 1.14)	5.59 (4.57; 6.75)	5.80 (4.75; 7.00)
Lung	0.14 (0.09; 0.22)	0.85 (0.56; 1.32)	0.88 (0.58; 1.37)
Lung adjusted (NS)	0.06 (0.04; 0.09)	0.37 (0.24; 0.56)	0.38 (0.25; 0.59)
Breast	0.08 (0.05; 0.13)	0.49 (0.29; 0.79)	0.51 (0.30; 0.82)

The risks are calculated for an age at exposure of 40 years, an attained age of 65 years using a mixed ERR and EAR model. The weights, w, for the mixed ERR and EAR (ERR: EAR) are: all solid cancer (0.5: 0.5), leukemia (0.5: 0.5), all solid cancer plus leukemia (0.5: 0.5) lung and lung adjusted for non-smoking (NS) (0.3: 0.7), breast (0: 1)

## Discussion

The application of RADS in radiation HRA for European astronauts has been presented here. This application involved the development and verification of a prototype software for calculating RADS, for any post mission age, for 4 cancer incidence outcomes related to hypothetical organ doses from exposures accumulated during up to 4 separate missions. However, up to now, only organ weighted doses and estimated mission effective doses have been explicitly considered in the calculations presented in this paper.

RADS is a convenient description of cumulative radiation risk that is independent of survival rates and has many other advantages (Ulanowski et al. 2019). RADS is less dependent than other quantities used in radiation protection, such as LAR and REIC, on large uncertainties involved with projections of contemporary demography and health population statistics far into the future. RADS is also, unlike REIC, independent of a knowledge of the underlying and highly uncertain survival curve. This latter feature makes RADS highly suitable for application to crews on international exploratory-class missions, requiring international risk alignments because the survival curves from the national statistics corresponding to the nationality of the astronaut’s space agency are not required. RADS closely resembles other quantities e.g., LAR and REIC at lower doses and, if the competing risks are negligible. RADS is not suggested here as a total replacement for conventional quantities, such as LAR, used to communicate general radiation risks for the purposes of radiation protection of general populations exposed to low doses. RADS has a rather more specific suitability, namely in applications for radiation risk assessments: for atypical groups or individuals such as astronauts; and at radiation organ doses higher than 1 Gy as received on some space missions and also in radiotherapy (with dose ranges up to several tens of Gy). These are the main reasons why RADS is highly suitable for and was chosen for the bespoke health risk assessment methodology for the radiation protection of astronauts based on European data described in this paper.

There are also some disadvantages inherent in RADS applications. Other quantities such as LAR and REIC are usually treated as being directly additive, post calculation, for the different outcomes of interest (e.g., as in ICRP 2007). In contrast, RADS is not additive, post calculation, for different outcomes. Although RADS can be defined, pre-calculation, for the sum of different outcomes to form a composite and then calculated for this composite outcome. RADS depends on age, but may be more heavily dependent on age at old ages than for middle ages, for cancers or diseases which tend to develop very late in life (e.g., lung cancer and cardiovascular disease). On the other hand, RADS is independent of competing risks which vary with, among other factors, gender and ethnicity, and tend to increase more at old age than at middle age.

The bespoke RADS based HRA for astronauts described here is based on outcome incidence rather than mortality because, among other reasons: cure rates for most diseases are increasing with time; survival times for persons with diseases are generally increasing; and astronauts post-mission undergo a higher level of medical screening than the general population, meaning that cancers are detected earlier and, therefore, have higher cure rates and lower mortality risks than for the general population of Europe on-average. Risks for the outcome incidence, while not directly quantifying the psychological effect of a cancer diagnosis and the resulting impact on quality of life and career progression, are a better choice in this respect than mortality outcomes which totally ignore these factors. If, however, mortality risks are actually required for decision making by flight directors, these can be readily obtained by either applying lethality factors to the incidence risks or by basing RADS on mortality risk models and mortality population data.

The overall choice for the selection of ERR and EAR models for input into the RADS calculations presented here was unfortunately highly constrained due to the necessity to choose models for which the data used to fit the models was publicly available so that the models could be re-fitted by the current authors to provide the unpublished EAR model for lung cancer and all covariance matrices that were an essential input requirement for the Monte-Carlo uncertainty analysis (Appendix A). In general, the choice was based on the most recent LSS cancer incidence models with publicly available data on the website of the Radiation Effects Research Foundation (http://www.rerf.or.jp). Several LSS ERR risk models for lung cancer were provided in Table 3 of Cahoon et al. (2017) for the most recent follow-up period (1958–2009). The choice was made to apply the simplest lung cancer ERR risk model i.e. The “radiation only” model, as opposed to the model also including a “generalized multiplicative” adjustment for radiation and smoking, was used to calculate the risks here. These two models have very similar central estimates for the ERR/Gy lung dose of 0.83 (95% CI 0.58; 1.09) and 0.81 (0.51; 1.18) for the radiation only and generalized multiplicative models, respectively and the radiation risk effect modifiers were also very similar in both models. Relative radiation risk for lung cancer was reported to be almost 4 times higher in men compared to women (Cahoon et al. 2017). In the newly fitted EAR model, unadjusted for smoking, the current authors do not find a statistically significant sex dependence which is reflected in the RADS results in Table 2. The large sex dependence in the ERR has been discussed as a limiting factor which may prevent female astronauts participating in space missions to the same extent as male astronauts on the basis of risk constraints for radiation protection. However, more work is needed by the RERF experts to provide a smoking adjusted EAR model before these preliminary results are confirmed. It is interesting to note that risk models for radiation-induced lung adenocarcinoma adjusted for smoking based on imputed data do not find a sex dependence in descriptive ERR models and in parameters of mechanistic models (Castelletti et al. 2019).

In the future, other risk models from occupational cohorts such as the Million Person Study (Boice et al. 2019) or as developed for the German probability of causation assessment tool ProZES (Ulanowski et al. 2020b) could also be implemented for astronaut HRA. Generally, it would be good practice to see how different risk models from different studies affect the HRA results, but given the stringent practical constraints mentioned above, this possibility was very limited at this point in time.

There are several ways to approach the setting of occupational radiation risk limits for astronauts and two tentative suggestions were given here. Ultimately, however, the space agencies will decide which approach to apply and useful approaches can be envisaged that involve the practically appealing and bespoke HRA for astronauts presented here.

The purpose of the HRA presented here is to delineate the risks at dose levels in Gy from space radiation. The risk calculations needed to be based on the use of colon, RBM, lung and breast, as the initial choice, of four target tissues for organ doses from A-bomb radiation used in the risks per unit dose based on the primary (LSS) data, and on the assumption that for applications in space, the LSS risks per organ dose in Gy is equal to the risk per effective dose in Sv. This assumption is based on a consideration that, for the very high energies involved in space radiation, the doses to these four tissues will, to first order, and for a given fluence, be equal. This assumption can only be made for space applications, because for ground-based applications there are clear differences in the LSS A-bomb gamma and neutron doses for particular organs, where an extreme example of this is the difference between mean neutron dose to the breast which is more than a factor of three times higher than the mean neutron dose to the colon, due to shielding by the human body (Kellerer et al. 2006). LSS neutron RBE dependence in the LSS risk models, upon which the RADS calculations are based, is accounted for here with an empirical LSS neutron RBE model (Hafner et al. 2021). This model allows the application of an LSS neutron RBE value of 80, as indicated by Cordova and Cullings (2019) and other comprehensive studies cited therein, rather than the RBE of 10 usually applied in most official analyses of the LSS data (e.g., Preston et al. 2007; Grant et al. 2017; Cahoon et al. 2017; Hsu et al. 2013). When all other factors are equal, the behaviour of decreasing cumulative risk estimates obtained using LSS risks per unit LSS doses calculated as the LSS neutron RBE increase from 10 to 80, allows longer space missions within given risk limits of radiological protection. Ideally, to better quantify this effect, the separate neutron and gamma doses for a selection of organs are required with all the publicly available LSS data for future work on astronaut HRA. However, the fact that some analyses indicate LSS neutron RBEs of 80 does not provide definitive evidence to imply that these values should be directly applied in space radiation dosimetry, especially, when the alternative values are based on experimentally derived data or measurements (e.g., as in ICRP 2013). Clearly, large uncertainties remain in differences in indicators of biological damaging effects from the gamma and neutron radiation delivered at 2–5 meV (that accounts for most of the dose in the LSS) and from the much higher energy radiation associated with galactic cosmic rays and solar particle events occurring in space. Conceivably such biological effects in space could differ by detrimental health outcome, but currently there is a paucity of published information available on this particular aspect.

All results presented here, except those for leukemia, are for a DDREF of 1, for the purposes of illustrating this HRA methodology. DDREF is recommended (ICRP 2007) for radiological protection purposes, to extrapolate from high doses and dose rates to lower values. However, because the doses to astronauts are not really low, it could be better to call this a DREF (dose rate effectiveness factor) in future work especially if values other than unity are applied.

Further work recommended to be done in the near future, apart from considering a wider range of organs and tissues at cancer risk than those considered here, involves two main aspects of extending the HRA framework presented here. First, a more detailed dosimetry needs to be investigated and applied for converting mission doses to the required weighted or equivalent organ doses for HRA. This phase should involve (a) some use of appropriately generalized/anonymised crew exposure data, (b) collaboration with dosimetry experts to carry out organ dose calculations for exposures in space, (c) consideration of knowledge from radiation biology, (d) input from space agency information technology experts, (e) a full consideration of the space dosimetry results given in ICRP (2013). ICRP (2013) is the basis of astronaut dose assessment and introduced the term effective dose equivalent. The effective dose equivalent is about one-third less than the effective dose in a mission to Mars. However, in paragraph 126 of ICRP (2013) it is stated that “An application of the quantity effective dose equivalent, however, is not recommended for the assessment of doses of individuals or small groups of astronauts when these should become a basis for risk estimates. …..; risk estimates should be based on either absorbed dose or dose equivalent data for the organs and tissues of males or females, and corresponding risk factors for these tissues for male and female adults”. Secondly, an investigation is required into how non-cancer effects may be appropriately included with cancer grouping outcomes, into the risk assessment software.

The inclusion of radiation effects on non-cancer outcomes for the immune system; respiratory system; endocrine system; eye lens opacification; the cardio-vascular system; and central nervous system, could be investigated and, where possible, included. However, this task will be far less straight-forward for non-cancer outcomes than it is for cancer outcomes. For example, radiation effects on heart diseases have been reported in the studies of the Hiroshima and Nagasaki A-bomb survivors (e.g., Takahashi et al. 2017) and at low dose rates in workers in nuclear facilities and high back-ground radiation areas, but the risks per unit dose vary widely and have large confidence intervals (see e.g., Table 4 in Shore et al. 2019; Hughson et al. 2017). Some epidemiological studies report a positive association of circulatory diseases with radiation exposure, but radiation effects and their mechanisms are not sufficiently investigated, although thought to involve vascular endothelial cell injury of arteries and vessels together with enhanced inflammatory reactions (Sylvester et al. 2018; Hughson et al. 2017). It is often difficult to locate appropriate population incidence statistics for non-cancer outcomes for inclusion into the risk calculations based on ERR models, and making further progress on this aspect could also be a priority.

Other aspects for further work already suggested (Walsh et al. 2019a) include long-term improvements to the new HRA method which could be extended to include considerations of developments in: innovative radiation track structure transport codes; further refinements to both A-bomb-relevant and space-relevant RBE models; results from nano-dosimetry; and radiobiological experiments, done to improve the characterisations (particle types, energy and energy deposition spectra, qualitative differences between high LET and HZE ions) of ionising radiation in space.

## Conclusion

This work done in this paper offers an alternative risk metric to be used for the assessment and communication of radiation-induced risks from space missions. Risks based on the outcome incidence rather than mortality are recommended for many different reasons discussed here. Risks based on RADS are recommended because RADS is (a) highly suitable for atypical groups not represented well by general population data and (b) based on fewer assumptions and input parameters than REIC. RADS is a very useful cumulative risk measure for astronaut HRA and is, unlike REIC, independent of a knowledge of the underlying and highly uncertain survival curve. Whereas REIC gives the probability of a premature incidence of a cancer (in the cancer grouping considered) attributable to radiation exposure, RADS gives a cumulative radiation risk conditional on survival until a certain age. RADS applied to disease incidence can be thought of as the cumulative decrease in the unknown probability of surviving disease (i.e., a particular cancer grouping) free up to a certain attained age, due to the radiation exposure at an earlier age. If, however, mortality risks are actually required for decision making by flight directors, these can also be readily obtained with this alternative methodology. RADS is also highly suitable for application on international missions requiring international risk alignments, because, unlike REIC, the survival curves from the national statistics corresponding to the nationality of the astronaut’s space agency, are not required. When all other factors are equal, the behaviour of decreasing cumulative risk estimates obtained using LSS risks per unit LSS doses calculated as the LSS neutron RBE increase from 10 to 80, allows longer space missions within given risk limits of radiological protection.

## Figures and Tables

**Fig. 5:**
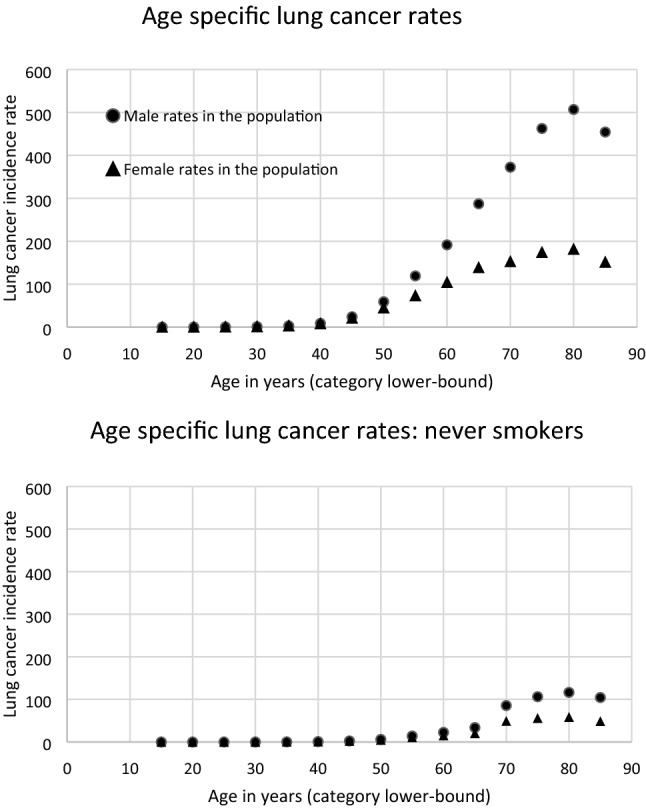
European CI5–8 country lung cancer age-specific incidence data (rates are in units of number of incident cases per 100,000 persons, in one year). The top panel shows the actual CI5–8 country data and the lower panel shows how these rates change after adjustment to non-smoking rates using the method described

## Appendix A Excess relative risk and excess absolute risk model fit-parameters and the parameter covariance matrices

Supplementary model fitting parameter results (Table 4) and parameter covariance matrices (Tables 5, 6, 7, 8, 9, 10, 11 and 12, rounded to the first 3 significant digits) are given here for re-fitting the LSS Excess Relative Risk (ERR) and Excess Absolute Risk (EAR) models for solid cancer, leukemia and breast cancer. The lung cancer incidence ERR model was also refitted but the EAR risk model applied in the software tool was fitted only by the current authors. This was required because the original publication (for follow-up 1958–2009, Cahoon et al. 2017) did not provide an *EAR* model without smoking adjustment (i.e., an *EAR* model with the same adjustments as the *ERR* model given as the first entry in Table 3 of Cahoon et al. (2017)). Such a model is analogous to the earlier *EAR* all solid cancer incidence model (for follow-up 1958–1998, Preston et al. 2007) with very similar fit parameters. The fit parameters (see Table 4) and parameter covariance matrix for this lung EAR model, unadjusted for smoking, were obtained by using the publicly available data set (rerf.or.jp) and the EPICURE software with the AMFIT module (Preston et al. 1993) for Poisson regression on grouped data. The other fit-parameters were refitted to the datasets used with the original LSS publications cited in the main text.

**Table 4:** Results of re-fitting a linear model to solid cancer, lung and breast cancer excess relative and absolute incidence as well as a linear-quadratic model to leukemia excess relative and absolute incidence to the atomic bomb data at attained age 70 years after exposure at age 30 years applying the EPICURE-AMFIT code to the atomic survivor data

Fit parameters for ERR					
	*β*^a^	*δ*^b^	*γ*_*a*_	*γ*_*e*_	*s*
Solid cancer	0.5024	–	− 1.567	− 0.2133	0.2864
Leukemia	0.7899	0.9501	− 1.09	− 0.8075	–
Lung cancer	0.8248	–	− 2.117	0.1486	0.5893
Breast cancer^c^	0.8776	–	− 2.224	0.004856	–

Fit parameters for EAR					
	*β*^d^	*δ*^e^	*γ*_*a*_	*γ*_*e*_	*s*
Solid cancer	53.31	–	2.35	− 0.3195	0.1385
Leukemia	1.059	1.086	− 1.447	0.4118	− 0.421^c^
Lung cancer	7.566	–	3.976	− 0.03775	0.1161
Breast cancer^c^	9.257	–	1.725	− 0.4543	–

^a^In Gy^−1^

^b^n Gy^−2^

^c^Women only

^d^In (10,000 PY Gy)^−1^

^e^In 10,000 PY^−1^ Gy^−2^

**Table 5:** Resulting covariance matrix for the ERR fit parameters from fitting a linear dose–response model to *solid cancer incidence* to the atomic bomb data at attained age 70 years after exposure at age 30 years applying the EPICURE-AMFIT code to the atomic survivor data with respect to weighted colon dose

ERR cov	*β*^a^	*γ*_*a*_	*γ*_*e*_	*s*
*β*^a^	0.00191	0.00282	0.00105	− 0.000275
*γ*_*a*_	0.00282	0.0557	− 0.00500	0.00163
*γ*_*e*_	0.00105	− 0.00500	0.00261	− 0.000309
*s*	− 0.000275	0.00163	− 0.000309	0.00345

^a^In Gy^−1^

**Table 6:** Resulting covariance matrix for the EAR fit parameters from fitting a linear dose–response model to *solid cancer incidence* to the atomic bomb data at attained age 70 years after exposure at age 30 years applying the EPICURE-AMFIT code to the atomic survivor data with respect to weighted colon dose

*EAR cov*	*β*^a^	*γ*_*a*_	*γ*_*e*_	*s*
*β*^a^	22.8	0.290	0.116	− 0.113
*γ*_*a*_	0.290	0.0440	− 0.00465	− 0.00236
*γ*_*e*_	0.116	− 0.00465	0.00259	− 0.000132
*s*	− 0.113	− 0.00236	− 0.000132	0.00387

^a^In (10,000 PY Gy)^−1^

**Table 7:** Resulting covariance matrix for the ERR fit parameters from fitting a linear-quadratic dose–response model to *leukemia incidence* to the atomic bomb data at attained age 70 years after exposure at age 30 years applying the EPICURE-AMFIT code to the atomic survivor data with respect to weighted RBM dose

ERR cov	*β*^a^	*δ*^b^	*γ*_*a*_	*γ*_*e*_
*β*^a^	0.218	− 0.0555	0.00466	0.0287
*δ*^b^	− 0.0555	0.123	0.0396	0.0242
*γ*_*a*_	0.00466	0.0396	0.197	− 0.0555
*γ*_*e*_	0.0287	0.0242	− 0.0555	0.0671

^a^In Gy^−1^

^b^In Gy^−2^

**Table 8:** Resulting covariance matrix for the EAR fit parameters from fitting a linear-quadratic dose–response model to *leukemia incidence* to the atomic bomb data at attained age 70 years after exposure at age 30 years applying the EPICURE-AMFIT code to the atomic survivor data with respect to weighted RBM dose

EAR cov	*β*^a^	*δ*^b^	*γ*_*a*_	*γ*_*e*_	*s*
*β*^a^	0.302	− 0.0746	0.0821	− 0.0204	− 0.0393
*δ*^b^	− 0.0746	0.175	0.0545	− 0.0114	− 0.0273
*γ*_*a*_	0.0821	0.0545	0.112	− 0.0288	− 0.00620
*γ*_*e*_	− 0.0204	− 0.0114	− 0.0288	0.0116	0.00162
*s*	− 0.0393	− 0.0273	− 0.00620	0.00162	0.0551

For males the column and row with the gender parameter $s$ can be neglected

^a^In (10,000 PY Gy)^−1^

^b^In 10,000 PY^−1^ Gy^−2^

**Table 9:** Resulting covariance matrix for the ERR fit parameters from fitting a linear dose–response model to *lung incidence* to the atomic bomb data at attained age 70 years after exposure at age 30 years applying the EPICURE-AMFIT code to the atomic survivor data with respect to weighted absorbed lung dose

ERR cov	*β*^a^	*γ*_*a*_	*γ*_*e*_	*s*
*β*^a^	0.0177	0.0283	− 0.00107	0.00000593
*γ*_*a*_	0.0283	0.895	− 0.0443	− 0.00833
*γ*_*e*_	− 0.00107	− 0.0443	0.0134	0.00123
*s*	0.00000593	− 0.00833	0.00123	0.0146

^a^In Gy^−1^

**Table 10:** Resulting covariance matrix for the EAR fit parameters from fitting a linear dose–response model to *lung cancer incidence* to the atomic bomb data at attained age 70 years after exposure at age 30 years applying the EPICURE-AMFIT code to the atomic survivor data with respect to weighted absorbed lung dose

EAR cov	*β*^a^	*γ*_*a*_	*γ*_*e*_	*s*
*β*^a^	2.00	0.295	− 0.0152	− 0.158
*γ*_*a*_	0.295	0.534	− 0.0413	− 0.0233
*γ*_*e*_	− 0.0152	− 0.0413	0.0132	0.00130
*s*	− 0.158	− 0.0233	0.00130	0.0361

^a^In (10,000 PY Gy)^−1^

**Table 11:** Resulting covariance matrix for the ERR fit parameters from fitting a linear dose–response model to *breast incidence* to the atomic bomb data at attained age 70 years after exposure at age 30 years applying the EPICURE-AMFIT code to the atomic survivor data with respect to weighted breast dose

*ERR cov*	*β*^a^	*γ*_*a*_	*γ*_*a*_
*β*^a^	0.0429	0.0879	0.00333
*γ*_*a*_	0.0879	0.494	− 0.0522
*γ*_*a*_	0.00333	− 0.0522	0.0186

^a^In Gy^−1^

**Table 12:** Resulting covariance matrix for the EAR fit parameters from fitting a linear dose–response model to *breast incidence* to the atomic bomb data at attained age 70 years after exposure at age 30 years applying the EPICURE-AMFIT code to the atomic survivor data with respect to weighted breast dose

EAR cov	*β*^a^	*γ*_*a*_	*γ*_*e*_
*β*^a^	2.49	0.274	0.0453
*γ*_*a*_	0.274	0.205	− 0.0346
*γ*_*e*_	0.0453	− 0.0346	0.0146

^a^In (10,000 PY Gy)^−1^

The model forms (ER—where ER is either ERR or EAR) give the risk per unit organ or tissue dose ${D}_{t}$ for all solid cancer, breast cancer and lung cancer, $\beta$, multiplied by a risk effect modifying function that depends on the variables attained age $a$, age at exposure $e$ and gender $s$:

4 $${\mathrm{ER}}_{\mathrm{t}}\left({D}_{\mathrm{t}},e,a,s\right)= \beta {D}_{\mathrm{t}}{\mu }_{\mathrm{t}}\left(e,a,s\right).$$

The subscript t denotes organ or tissue and ${\mu }_{\mathrm{t}}$ is the risk effect modifying function:

5 $${\mu }_{\mathrm{t}}\left(e,a,s\right)=\mathrm{exp}\left({\gamma }_{e}\left(\frac{e-30}{10}\right)+{\gamma }_{a}\mathrm{log}\left(\frac{a}{70}\right)\right)\left(1\mp s\right),$$

where − is used for males and + for females. The fit parameters ${\gamma }_{e}$, ${\gamma }_{a}$ and $s$ are sex-averaged and centred at an attained age $a$ of 70 years and an age at exposure $e$ of 30 years.

The leukemia models have a linear-quadratic dose–response model:

6 $${\mathrm{ERR}}_{\mathrm{L}}\left({D}_{\mathrm{m}},e,a,s\right)=\left( \beta {D}_{\mathrm{m}}+\delta {{D}_{\mathrm{m}}}^{2}\right){\mu }_{\mathrm{L}1}\left(e,a\right),$$

7 $${\mathrm{EAR}}_{\mathrm{L}}\left({D}_{\mathrm{m}},e,a,s\right)=( \beta {D}_{\mathrm{m}}+\delta {D}_{\mathrm{m}}^{2}){\mu }_{\mathrm{L}2}(e,a,s)$$

where $\beta$ and $\delta$ are the linear and quadratic dose–response parameters. L denotes leukemia, ${D}_{\mathrm{m}}$ red bone marrow dose and ${\mu }_{\mathrm{L}1}$ and ${\mu }_{\mathrm{L}2}$ are the risk effect modifying functions:

8 $${\mu }_{\mathrm{L}1}\left(e,a\right)=\mathrm{exp}\left({\gamma }_{e}log\left(\frac{a-e}{40}\right)+{\gamma }_{a}\mathrm{log}\left(\frac{a}{70}\right)\right),$$

9 $${\mu }_{\mathrm{L}2}\left(e,a,s\right)=\mathrm{exp}\left({\gamma }_{e}\left(\frac{e-30}{10}\right)+{\gamma }_{a}\mathrm{log}\left(\frac{a}{70}\right)\right)\mathrm{exp}\left(s\right).$$

The fit parameters ${\gamma }_{e}$ and ${\gamma }_{a}$ are sex-averaged and centred at an attained age $a$ of 70 years and an age at exposure $e$ of 30 years. The fit parameter $s$ is given for women and set to 0 for men.

## Appendix B Never smoker age-specific lung cancer rate estimation

The idea is to take the general population [made up from current smokers (CS), never smokers (NS), and former smokers (FS)] based age-specific lung cancer incidence rates for the CI5–8 countries and approximately adjust them to represent the rates for never smokers as most relevant for astronaut health risk assessment. The NASA methodology in Chappell (2014) has been adopted and adapted here and applied, to European CI5–8 country lung cancer age-specific incidence data (Fig. 5 top panel).

The steps required to estimate never smoke lung rates by gender and age:

1. Obtain relative risks for lung cancer mortality, ${\mathrm{RR}}_{\mathrm{M}}$ for current (CS) and former smokers (FS) in contemporary cohorts in comparison to lifetime never smokers (NS). ${\mathrm{RR}}_{\mathrm{M}}= {\mathrm{RR}}_{\mathrm{CS}} \mathrm{or} {\mathrm{RR}}_{\mathrm{FS}}$ are the assumed relative risk for lung cancer mortality associated with smoking exposure in CS and FS relative to never smokers. (Thun et al. 2013): “For women who were current smokers, as compared with women who had never smoked, the relative risks of death from lung cancer were 2.73, 12.65, and **25.66** in the 1960s, 1980s, and **contemporary cohorts**, respectively; corresponding relative risks for male current smokers, as compared with men who had never smoked, were 12.22, 23.81, and **24.97**.” where the bold has been added to indicate the values applied here.
2. Convert these mortality risks into incidence risks:
  10 $${\text{RR}}_{{\text{M}}} = {\text{RR}}_{{\text{I}}} \times L,$$
  where the lethality factor, $L$ for lung cancer of 0.89 for males and females (ICRP 2007) is applied.
3. Obtain estimates of smoking prevalence, $p$, for the populations and years and age categories of interest. ${p}_{\mathrm{i}}$ is the prevalence of never smokers (NS), former smokers (FS), and current smokers (CS) in the German population for 2012 (Zeiher et al. 2018, Figs. 1 and 2, read from graphics). There is no direct information for FS in age categories in Zeiher et al. (2018), but these can be inferred from the data provided in the paper. Overall, the prevalence percentages for NS, FS and CS are 38, 30, 32 for males and 53, 22, 25 for females, respectively. This assumes that prevalence percentages in the German population are a good approximation for prevalence in the 8 European countries, which the authors were unable to locate in published form for each country and age group.
4. Estimate the relative risk for lung cancer incidence associated with mixed exposure of smokers, former smokers and never smokers relative to never smokers (by sex and age group). The ${\mathrm{RR}}_{\mathrm{I}}$ can be calculated as follows:
  11 $${\text{RR}}_{{\text{I}}} = p_{{{\text{NS}}}} + p_{{{\text{FS}}}} \left( {L \times {\text{RR}}_{{{\text{FS}}}} } \right) + p_{{{\text{CS}}}} \left( {L \times {\text{RR}}_{{{\text{CS}}}} } \right).$$
5. Obtain the fraction that is not attributable to smoking, never smoking attributable fraction (NASAF),
  12 $${\text{NSAF}} = 1 - {\text{SAF,}}$$
  where the smoking attributable fractions (SAFs) by sex and age group:
  13 $$\mathrm{SAF}=\frac{{\mathrm{RR}}_{\mathrm{I}}-1}{{\mathrm{RR}}_{\mathrm{I}}},$$
  14 $$\mathrm{NSAF}= 1-\frac{\left({\mathrm{RR}}_{\mathrm{I}}-1\right)}{{\mathrm{RR}}_{\mathrm{I}}}=\frac{1}{{\mathrm{RR}}_{\mathrm{I}}}.$$
6. The NSAF then multiplies the sex and age-specific CI5–8 country lung cancer rates to estimate NS lung rates by gender and age (Fig. 5 lower panel).
